# Effect of regular exercise on stroke prevention: an instrumental variables approach

**DOI:** 10.1186/s12889-025-22298-y

**Published:** 2025-03-27

**Authors:** Wonseok Jeong

**Affiliations:** Department of Public Health Sciences, Graduate School of Public Health, Seoul National University, 1 Gwanak-Ro, Gwanak-Gu, Seoul, 08826 Republic of Korea

**Keywords:** Stroke, Regular exercise, Instrument variable, Proximity to exercise facilities

## Abstract

**Aims:**

In South Korea, around 105,000 individuals experience strokes annually, with over 26,000 deaths and the incidence rate is keep rising due to the aging population. Physical inactivity is a major global health issue, and regular exercise is known to prevent many diseases, including stroke. Yet, previous observational studies may be biased due to unobserved factors influencing exercise and stroke occurrence. Therefore, this study aims to examine an impact of regular physical activity on stroke prevention among South Korean adults using instrumental variable approach in order to avoid problems with reverse causality and other unobserved confounding factors.

**Methods:**

Data were obtained from the 2014 and 2016 Korean Community Health Surveys of 416,032 Korean adults. Regular exercise was the main independent variable and proximity to exercise facilities is used as an instrument. The dependent variable, presence of stroke, was defined by a doctor’s diagnosis. Probit regression analyses were performed to examine the associations between the independent variable and both dependent variable and an instrument. Causal effect of regular exercise on stroke prevention were estimated with the bivariate probit regressions using proximity to exercise facilities as an instrument for regular exercise.

**Results:**

Consistent with prior knowledge, proximity to exercise facilities positively influenced regular exercise. The results from both multivariable probit and bivariate probit models indicate that engaging in regular exercise has a strong negative effect on the incidence of stroke. The estimated effects of regular physical activity on stroke prevention range from -0.174 percentage points (ATE) to -0.154 percentage points (ATT) in the bivariate probit model.

**Conclusion:**

This study makes an important contribution by providing IV estimates on the effects of conducting regular exercise on stroke prevention based on a representative sample of South Korean adults. My findings suggest that conducting regular exercise reduces the risk of stroke even after accounting for the potential reverse causality and unobserved related factors.

## Introduction

When a stroke occurs, brain cells are deprived of the necessary oxygen, potentially leading to disabilities such as difficulty with movement, speech, or cognition, and in severe cases, death [1]. In South Korea, approximately 105,000 individuals experience a new or recurrent stroke each year, with over 26,000 stroke-related deaths reported in 2010 [2]. More importantly, due to the rapid growth of Korea's elderly population, stroke incidence is anticipated to keep escalate [3, 4]. The crude incidence rate of stroke remained around 200 per 100,000 person-years from 2011 to 2015, but it surged to 218.4 per 100,000 person-years in 2019 [5]. To address this growing burden of stroke, it is crucial to significantly improve the control and identification of stroke risk factors.

Physical inactivity is recognized as a significant global health issue in the modern era. There is an increasing number of inactive individuals in various nations, which poses risks to personal health, healthcare utilization, and overall public healthcare expenses [6]. Undeniable evidence supports the effectiveness of regular physical activity in preventing numerous diseases, including diabetes, hypertension, osteoporosis, and even premature death [7]. A previous study examining the causal relationship between physical activity and chronic disease development found that engaging in vigorous physical activity is associated with a modest reduction in the risk of major coronary heart disease [8]. Similarly, another study reports that increased physical activity lowers blood pressure in individuals with hypertension, raises high-density lipoprotein (HDL) cholesterol in a dose–response manner, and reduces the incidence of diabetes [9]. Consequently, initiatives promoting exercise and intervention programs aimed at boosting individual physical activity levels are consistently prioritized by health policymakers worldwide [10]. Meanwhile, numerous observational studies have identified that physical activity also plays a significant role in stroke prevention [11, 12]. Engaging in regular moderate to vigorous intensity physical activity reduces the risk of a first-ever stroke by 16% among Korean adults.

Yet, the findings from earlier studies have potential issues due to unobserved factors linked to both an individual's decision to exercise and the occurrence of stroke. The likelihood of engaging in physical activity is strongly associated with undisclosed health-related factors such as an individual's perspective on health, current health status, and healthy lifestyle choices. These factors are positively correlated with sports activity and negatively correlated with health issues. Yet, such health conditions of individuals cannot be fully accounted for in observational studies, leading to omitted variable bias [6]. Also, the impact of physical activity on stroke is subject to reverse causality, when correlational studies cannot establish causality. For instance, there might be a negative correlation between stroke incidence and physical activity because individuals who have had a severe stroke may not be able to engage in regular exercise. More importantly, given the extensive body of research demonstrating the positive effects of physical activity on the health of stroke survivors, individuals with milder strokes may be more likely to adopt a more active lifestyle than before [13–15]. This behavioral response introduces heterogeneity in the sample, potentially leading to an underestimation of the true effect of physical activity on stroke prevention in observational studies. Notably, the current evidence on the relationship between physical activity and stroke prevention is derived exclusively from observational studies, which limits the ability to establish causality.

All issues related to heterogeneity, omitted variables, omitted variable bias, and reverse causality can be addressed using instrumental variable (IV) techniques, provided that a series of critical assumptions are satisfied [16]. In the present study, I use instrumental variable (IV) estimation, using proximity to sports facilities as an instrument for physical activity. Several previous studies have demonstrated the relationship between physical activity and the proximity of exercise facilities across different population groups [13, 17, 18]. Furthermore, the presence of a stroke is unlikely to be a consequence of an individual's proximity to health facilities, except through the influence of exercise, thereby meeting the critical assumptions. This approach of using proximity to sports facilities as an instrument has previously been used in studies of subjective well-being, healthcare utilization, and health [19, 20].

Therefore, this study aims to examine an impact of regular physical activity on stroke among South Korean adults using a quasi-experimental approach. I use instrumental variable (IV) estimation to distinguish health benefit of physical activity from unobserved heterogeneity.

## Material and methods

### Data and study participants

Data for this study were sourced from the 2014 and 2016 Korea Community Health Survey (KCHS) conducted as two separate cross-sectional surveys. The KCHS is an annual national survey designed to assess public health status and behaviors at the community level since 2008. The year 2015 was not included in this study due to the absence of the instrument variable (IV) questionnaire in that year's survey. Managed by the Korea Centers for Disease Control and Prevention, this survey targets adults aged 19 and older, using interviews from 255 communities across Korea. The survey employs stratified systematic sampling to select sample areas and a systematic two-step sampling method to choose sample households, ensuring the sample accurately represents the entire population [21, 22]. The KCHS protocol received approval from the Institutional Review Board of Korea Centers for Disease Control and Prevention (2016–10-01-P-A), and written informed consent was acquired from all participants of the KCHS. Further details about the KCHS can be found in other publications [23].

Out of the 457,164 individuals who participated in the 2014 and 2016 surveys, 451,872 adults remained after excluding those under 19 years old. Next, 68 individuals with missing dependent variable data and 3,361 individuals lacking information on the instrumental variable (IV) or independent variables were removed. Finally, participants with missing covariates, such as smoking status, drinking status, and subjective health status, were excluded. This resulted in a final sample size of 416,239.

### Variables

The primary independent variable was regular exercise. Regular moderate- or high-intensity exercise was categorized according to the WHO's physical activity guidelines, which recommend more than 150 min per week of moderate-intensity exercise or more than 75 min per week of high-intensity exercise [24]. Participants provided responses to the questions, “On the days when moderate-intensity exercise was performed, how long did it last on average?” and “On the days when high-intensity exercise was performed, how long did it last on average?”. Using these responses, the average durations of each type of exercise were calculated. These durations were then multiplied by responses to the questions, “How many days a week did you perform moderate/high-intensity exercise?”. Based on this information, the average total durations of moderate/high-intensity exercise performed per week were computed, and participants were categorized into “yes” or “no” groups accordingly.

The demographic characteristics considered in the study included participants' age groups (20 − 39 years, 40 − 59 years, and ≥ 60 years) and sex. Socioeconomic factors encompassed the participants' education level (categorized as middle school or lower, high school, and college or higher), marital status (married, separated or divorced, or never married), region (urban area, rural area), and their occupational classification. Occupations were classified according to the Korean version of the Standard Classification of Occupations: white-collar (office work), pink-collar (sales and services), blue-collar (forestry, fishery, armed forces, and agriculture), and unemployed [25]. The region was categorized into urban and rural areas depending on whether it was a metropolitan city.

Health-related characteristics included drinking status (heavy drinking, moderate drinking, and light drinking) by average number of days drinking (≥ 2 days/week, ≤ 1 day/week, and 1 day/month, respectively), smoking status (yes, no), body mass index (BMI [overweight or normal]), diabetes (yes, no) and hypertension (yes, no). Those who reported to currently smoke any type of cigarettes were classified as “yes”. BMI ≥ 25 kgm^2^ was used as the cut-off point in this study, following the WHO definition for obesity in Asian adults [26]. Diabetes and hypertension were defined depending on answers to the questions, “Have you ever been diagnosed with diabetes/hypertension by a doctor?”.

The primary dependent variable in this study was the presence of stroke. Participants were classified as stroke patients if they answered "yes" to either of the following questions: "Have you ever been diagnosed with stroke by a doctor?" or "Are you currently receiving medical treatment for stroke?".

In my statistical model for stroke prevention, I use proximity to exercise facilities as an instrumental variable (IV) for regular physical activity. Participants were categorized into “yes” or “no” groups based on their responses to the question, “How difficult was it to find places to exercise near where you live within the past year?” Those who answered “very easy” or “relatively easy” were placed in the “yes” group, while those who answered “very hard” or “relatively hard” were placed in the “no” group. My IV strategy relies on the premise that closer proximity to exercise facilities likely encourages more frequent exercise (instrument relevance), but should not directly affect the incidence of stroke, given my controls for health, activity, occupational risk factors, and other relevant socio-demographic characteristics.

### Statistical analysis

Chi-square tests were used to analyze the general characteristics of the study population. Probit regression analyses were performed to examine the association between my main independent variable, dependent variable, and instrument and after accounting for potential confounding variables including demographic, socioeconomic, and health-related characteristics. I then used multivariable bivariate probit model for my causal IV analysis investigating the impact of physical activity on the presence of stroke. Lastly, due to the high age dependency of stroke, I conducted additional robustness check relative to my main models with different range of population groups (< = 40, 70 <). For the bivariate probit models, along with the coefficients, I also report the average treatment effect (ATE) and average treatment effects on the treated (ATT), which represents the impact of physical activity on stroke among the entire population and among those who actually engaged in regular exercise, respectively [27]. Following the simulation evidence from the previous study, I bootstrapped the standard errors of the ATEs and ATTs using 500 repetitions to ensure robustness [28]. Results are reported with 95% confidence interval (CI) and differences were considered statistically significant with a *p*-value < 0.05. All data analyses were conducted using R (http://www.r-project.org) and its supported packages.

## Results

Table 1 shows the general characteristics of the study population. The data included 8,974 participants with stroke and 407,058 participants without stroke, out of a total of 416,032 participants. 93,603 (22.5%) participants conducted regular exercise while 322.429 (77.5%) did not. The Chi-square test showed that these differences were statistically significant.

**Table 1:** General characteristics of study population (*n* = 416,239)

Variables	Total		Stroke				
			**Yes**		**No**		***P*-value**
**Total**	416,239	(100.0)	9,002	(2.2)	407,237	(97.8)	
**Regular Exercise**							< .0001
Yes	93,631	(22.2)	1,336	(1.4)	92,295	(98.6)	
No	322,608	(75.7)	7,666	(2.4)	314,942	(97.6)	
**Sex**							< .0001
Male	183,646	(45.2)	4,558	(2.4)	183,646	(97.6)	
Female	223,591	(54.8)	4,444	(1.9)	223,591	(98.1)	
**Age (years)**							< .0001
≥ 60	143,569	(36.3)	7,660	(5.1)	143,569	(94.9)	
40–59	161,549	(39.1)	1,267	(0.8)	161,549	(99.2)	
20–39	102,119	(24.6)	75	(0.1)	102,119	(99.9)	
**Educational level**							< .0001
Middle school or less	145,496	(36.6)	6,665	(4.4)	145,496	(95.6)	
High school	117,258	(28.5)	1,564	(1.3)	117,258	(98.7)	
College or over	144,483	(34.9)	773	(0.5)	144,483	(99.5)	
**Drinking Status**							< .0001
Heavy drinking	193,090	(48.0)	6,633	(3.3)	193,090	(96.7)	
Moderate drinking	181,437	(44.0)	1,693	(0.9)	181,437	(99.1)	
Light drinking	32,710	(8.0)	676	(2.0)	32,710	(98.0)	
**Occupational classification**							< .0001
White-collar	81,830	(19.7)	238	(0.3)	81,830	(99.7)	
Blue-collar	128,275	(31.3)	2,163	(1.7)	128,275	(98.3)	
Pink-collar	52,242	(12.6)	390	(0.7)	52,242	(99.3)	
None	144,890	(36.3)	6,211	(4.1)	144,890	(95.9)	
**Marital Status**							< .0001
Married	282,494	(69.3)	5,965	(2.1)	282,494	(97.9)	
Separated or divorced	65,514	(16.4)	2,881	(4.2)	65,514	(95.8)	
Unmarried	59,229	(14.3)	156	(0.3)	59,229	(99.7)	
**Smoking**							< .0001
Yes	154,682	(37.2)	4,060	(2.6)	150,622	(97.4)	
No	261,557	(62.8)	4,942	(1.9)	256,615	(98.1)	
**Region**							< .0001
Urban area	103,024	(32.9)	1,643	(1.6)	101,381	(98.4)	
Rural area	313,215	(67.1)	7,359	(2.3)	305,856	(97.7)	
**Diabetes**							< .0001
Yes	39,679	(10.2)	2,580	(6.1)	39,679	(93.9)	
No	367,558	(89.8)	6,422	(1.7)	367,558	(98.3)	
**Hypertension**							< .0001
Yes	101,054	(25.8)	6,201	(5.8)	101,054	(94.2)	
No	306,183	(74.2)	2,801	(0.9)	306,183	(99.1)	
**BMI**							< .0001
Obesity	104,621	(25.7)	2,388	(2.2)	104,621	(97.8)	
Normal	302,616	(74.3)	6,614	(2.1)	302,616	(97.9)	

*BMI* body mass index

Table 2 presents the first-stage probit regression results when ‘regular exercise’ was used as a dependent variable. In consistent with the general expectations, those living close to the exercise facilities are more likely to conduct regular exercise than those living far from the facilities. Men exercised more than women, while heavy drinkers and moderate drinkers exercised less than the light drinkers. The first-stage F-statistics of the joint significance of the instruments were used to assess their relevance. According to the rule of thumb by Staiger and Stock, the F-statistics should exceed 10 [29]. In this study, the first-stage F-statistic was above 10, indicating that the instrumental variable is relevant.

**Table 2:** First-stage regression results for IV

Variables	Coef	SE	*P*-value
**Proximity to Exercise Facilities**			
Yes	0.046	0.00524	< .0001
No	Ref		
**Sex**			
Male	0.149	0.00666	< .0001
Female	Ref		
**Age (years)**			
≥ 60	0.042	0.00899	< .0001
40–59	0.072	0.00690	< .0001
20–39	Ref		
**Educational level**			
Middle school or less	−0.073	0.00771	< .0001
High school	−0.050	0.00624	< .0001
College or over	Ref		
**Drinking Status**			
Heavy drinking	−0.011	0.00859	< .0001
Moderate drinking	−0.06	0.00832	< .0001
Light drinking	Ref		
**Occupational classification**			
White-collar	0.072	0.00713	< .0001
Blue-collar	0.430	0.00582	< .0001
Pink-collar	0.228	0.00752	< .0001
None	Ref		
**Marital status**			
Married	0.050	0.00758	< .0001
Separated or divorced	−0.150	0.00681	< .0001
Unmarried	Ref		
**Smoking**			
Yes	−0.063	0.00656	< .0001
No	Ref		
**Region**			
Rural area	0.053	0.00524	< .0001
Urban area	Ref		
**Diabetes**			
Yes	−0.057	0.00787	< .0001
No	Ref		
**Hypertension**			
Yes	−0.040	0.00580	< .0001
No	Ref		
**BMI**			
Obesity	0.022	0.00508	< .0001
Normal	Ref		

*Coef* Coefficient, *SE* Standard Error

Table 3 presents the associations and causal links between regular exercise and stroke prevention using multivariable probit and IV probit models. My estimation results from both models indicate that regular exercise has a negative effect on the presence of stroke conditional on all other controls. Those engaging in regular exercise were less likely to have strokes compared to those do not regardless of the model specification. Yet, the preventive impact of regular exercise presented to be much stronger in the IV probit compared to that of the probit model (probit: Coef. = −0.100, SE = 0.013; bivariate probit: Coef. = −5.651, SE = 0.646). For the bivariate probit models, along with the coefficients, I also report ATE and ATT. The ATE indicates that the preventive effect of exercise on stroke across the entire population is estimated to be −17.4 percentage points. Similarly, the IV probit model shows an ATT of −15.4 percentage points, meaning that among those who actually engaged in regular exercise, the preventive impact on stroke is estimated to be 15.4 percentage points. All results were statistically significant.

**Table 3:** Results of the probit and bivariate probit regression

Variables	Probit			^a^Bivariate Probit		
	**Coef**	**SE**	**P-value**	**Coef**	**SE**	*P*-**value**
**Regular Exercise**						
Yes	−0.086	0.013	< .0001	−6.219	0.596	< .0001
No	Ref			Ref		
**Sex**						
Male	0.308	0.017	< .0001	0.565	0.029	< .0001
Female	Ref			Ref		
**Age (years)**						
≥ 60	0.905	0.042	< .0001	0.975	0.042	< .0001
40–59	0.559	0.040	< .0001	0.697	0.043	< .0001
20–39	Ref			Ref		
**Educational level**						
Middle school or less	0.245	0.019	< .0001	0.114	0.023	< .0001
High school	0.084	0.020	0.4231	−0.004	0.021	0.8417
College or over	Ref			Ref		
**Drinking Status**						
Heavy drinking	0.280	0.019	0.4231	0.063	0.028	0.0236
Moderate drinking	0.016	0.020	< .0001	−0.106	0.023	< .0001
Light drinking	Ref			Ref		
**Occupational classification**						
White-collar	−0.467	0.027	< .0001	−0.370	0.029	< .0001
Blue-collar	−0.352	0.012	< .0001	0.426	0.074	< .0001
Pink-collar	−0.33	0.022	< .0001	0.048	0.042	0.2587
None	Ref			Ref		
**Marital Status**						
Unmarried	−0.049	0.035	0.168	0.429	0.036	0.239
Separated or divorced	0.046	0.012	< .0001	−0.181	0.025	< .0001
Married	Ref			Ref		
**Smoking**						
Yes	0.128	0.016	< .0001	0.039	0.018	0.029
No	Ref			Ref		
**Region**						
Rural area	0.051	0.013	< .0001	0.127	0.018	< .0001r
Urban area	Ref			Ref		
**Diabetes**						
Yes	0.172	0.012	< .0001	0.081	0.015	< .0001
No	Ref			Ref		
**Hypertension**						
Yes	0.046	1204.000	< .0001	0.383	0.012	< .0001
No	Ref			Ref		
**BMI**						
Obesity	0.005	0.011	0.6438	0.046	0.012	< .0001
Normal	Ref			Ref		

*Coef* Coefficient, *SE* Standard Error

^a^ATE, Average Treatment Effect: −0.167 (CI: −0.289, −0.143), ATT, Average Treatment Effect on the Treated: −0.171 (CI: −0.303, −0.135)

Table 4 presents the robustness checks for my estimates of the causal effect of exercise on stroke prevention with regard to three alternative specifications of my empirical models. First, I re-estimated the multivariable probit and bivariate probit models using a different age group that is more susceptible to stroke. The estimation results remained largely unchanged even when focusing on individuals aged 40 to 70 (probit: Coef. = −0.064, SE = 0.014; bivariate probit: Coef. = −7.571, SE = 1.003). As a second robustness check, I explore the potential heterogeneity of the causal effect of exercise on stroke prevention across different regions. This analysis is crucial because individuals living in urban areas are more likely to have better access to exercise facilities, which could bias the results if not properly accounted for. The results remained robust, with statistically significant effects observed in both urban and rural areas, suggesting that the preventive impact of exercise on stroke is not driven by region-specific factors.

**Table 4:** Robustness analysis: results of the probit and bivariate probit regression

Variables	Probit			Bivariate Probit		
	**Coef**	**SE**	**P-value**	**Coef**	**SE**	*P***-value**
**Using Age Group More Susceptible to Stroke**						
**40–70**						
Regular Exercise	−0.064	0.014	< .0001	−7.571	1.003	< .0001
No	Ref			Ref		
**Using Group from Different Region**						
**Rural area**						
Regular Exercise	−0.090	0.051	< .0001	−4.948	0.726	< .0001
No	Ref			Ref		
**Urban area**						
Regular Exercise	−0.134	0.032	< .0001	−3.607	0.856	< .0001
No	Ref			Ref		
**Using Continuous Measures of Physical Activity**						
**Exercise duration**						
Moderate-Intensity Exercise (hours)	−0.013	0.002	< .0001	−1.886	0.212	< .0001
High-Intensity Exercise (hours)	−0.02	0.003	< .0001	−1.688	0.190	< .0001

Every model includes controls for sex, age, educational level, drinking status, occupational classification, smoking, BMI, region, marital status, hypertension, diabetes

*Coef* Coefficient, *SE* Standard Error

Lastly, I examine the impact of replacing the main explanatory variable, regular exercise, with continuous measures to address the potential loss of information associated with the use of a dummy variable. Specifically, I re-estimated the models using two continuous variables: the duration of moderate-intensity exercise in hours and the duration of high-intensity exercise in hours. Although the coefficients in both models were slightly lower compared to those from the main estimations, which can be attributed to the majority of respondents not engaging in regular exercise, the results from both the probit and bivariate probit models remained statistically significant. Notably, the bivariate probit models demonstrated a stronger preventive effect, consistently indicating the presence of heterogeneity and the necessity of instrumental variable models, in line with the findings from the main models.

## Discussion

This study examines the causal effect of regular physical activity on stroke prevention among South Korean adults using an instrumental variable approach. The findings indicate that engaging in regular physical activity significantly reduces the likelihood of stroke, with estimated effects ranging from 15.4 to 43.9 percentage points, depending on the model specification. These results provide strong evidence that physical activity plays a crucial role in reducing stroke risk, even after accounting for potential endogeneity concerns. With the rapid increase in Korea's elderly population, the incidence of stroke is expected to rise, emphasizing the need for targeted prevention strategies. While previous studies have largely relied on observational methods, they often overlook the endogenous relationship between physical activity and stroke. By leveraging data from the 2014 and 2016 KCHS and incorporating key demographic, socioeconomic, and health-related variables, this study provides a more robust estimation of this relationship.

In addition to prevention, engaging in regular physical activity is also associated with reduced severity of stroke in the future. According to a previous study, higher levels of pre-stroke physical activity were associated with a 39–88% reduction in acute infarct growth (measured within 24 h after stroke admission) and smaller final infarct volumes (measured one month post-stroke) in 102 ischemic stroke patients [30]. Although the mechanisms mediating the reduction in stroke risk are not fully understood, substantial evidence suggests that physical activity influences stroke risk factors. In fact, increased physical activity is associated with several potential neuroprotective mechanisms [31]. The beneficial effects of physical activity on stroke-related cerebral vascular function involve a complex interplay of neurogenesis and angiogenesis that promotes regenerative and repair mechanisms in the human brain [32]. Individuals with higher levels of physical activity prior to a stroke experienced a greater increase in vascular endothelial growth factors (VEGF) from baseline to 7 days post-stroke. This increase in VEGF was associated with better functional outcomes (Modified Rankin Scale (mRS) ≤ 2) at 3 months follow-up and smaller final infarct size [33]. Given that VEGF plays important roles in neuroprotection, angiogenesis, and post-ischemic neuronal repair, physical activity may contribute to neuroprotection, and thereby stroke prevention, through the upregulation of VEGF expression [34]. Beyond its neuroprotective effects, physical activity reduces stroke risk through multiple physiological pathways. Exercise improves cardiovascular health by reducing arterial stiffness, enhancing endothelial function, and lowering blood pressure, all of which reduce cerebrovascular risk [35]. Additionally, it regulates immune responses by lowering pro-inflammatory cytokines such as C-reactive protein (CRP) and tumor necrosis factor-alpha (TNF-α), while increasing anti-inflammatory markers like interleukin-10 (IL-10), which contribute to vascular protection [36]. These mechanisms reinforce the systemic benefits of exercise in stroke prevention.

By using an instrument variable approach, I could arguably provide better estimates of the relationship between exercise and stroke than could have done without an instrument. Nevertheless, instrumental variable analyses are based on the strength of the association between the instrument and the exposure, and on several key assumptions [37]. Both my data and previous studies present a strong association between proximity to exercise facility and exercise status, but the essential assumption that the instrument should only influence the outcome through the exposure of interest is difficult to evaluate [38, 39]. Nevertheless, I argue that the exogeneity condition of my instrument is plausible for the following reasons. First of all, sports facilities are provided by communities and not by individuals. The proximity to exercise facility is not chosen by the residents, but exogenously decided. Demographic and socioeconomical variables such as age, educational level, occupational classification, which indirectly reflect individuals’ characteristics on residential choices are also adjusted to further enhance the exogeneity.

My study has some limitations. Participants of the KCHS were interviewed face-to-face, which helped build trust and rapport between interviewers and respondents, but it might have also led interviewees to underreport behaviors that are not socially acceptable Furthermore, due to the absence of necessary survey questions in recent surveys, the dataset I used is somewhat outdated and may not fully reflect the current situation. Despite these limitations, the following strengths of the study were observed. First, the KCHS is conducted by a national institution; thus, the data obtained from it are more reliable and representative of the entire Korean population than those obtained by private institutions. Second, this study is one of the few to identify the causal impact of regular exercise on stroke prevention using an instrument variable approach. Third, 'proximity to sports facilities' was based on subjective perception rather than the actual distance from the facilities. Since the same distance can be perceived differently by different individuals—being too far for some and acceptable for others—the subjective perception serves as a better instrument.

## Conclusions

This study provides evidence that regular physical activity significantly reduces the likelihood of stroke, reinforcing its role as a key preventive measure, particularly for the aging Korean population. Using a quasi-experimental approach, the findings establish a causal relationship between physical activity and stroke prevention, addressing limitations in prior observational studies. Given the increasing burden of stroke in Korea, public health policies should prioritize promoting regular exercise through targeted interventions and awareness programs. Future research should explore long-term effects and assess how different forms of physical activity contribute to stroke risk reduction.

## Data Availability

All K-CHSI data may be downloaded from the KCHS site (https://chs.kdca.go.kr/chs/rdr/rdrInfoProcessMain.do). The entire data file may be downloaded without logging in by choosing the button labeled “파일다운로드” (i.e., “File download”). Data files are provided in Excel. There are a total of 4 files, from version 1.0 to 1.3. In addition, the website provides a search system that allows users to select a region and year for a variable of interest, then save their search results.
